# Reproducibility of *In Vivo* Corneal Confocal Microscopy Using an Automated Analysis Program for Detection of Diabetic Sensorimotor Polyneuropathy

**DOI:** 10.1371/journal.pone.0142309

**Published:** 2015-11-05

**Authors:** Ilia Ostrovski, Leif E. Lovblom, Mohammed A. Farooqi, Daniel Scarr, Genevieve Boulet, Paul Hertz, Tong Wu, Elise M. Halpern, Mylan Ngo, Eduardo Ng, Andrej Orszag, Vera Bril, Bruce A. Perkins

**Affiliations:** 1 Lunenfeld-Tanenbaum Research Institute, Mount Sinai Hospital, Toronto, Ontario, Canada; 2 Division of Endocrinology and Metabolism, Department of Medicine, University of Toronto, Toronto, Ontario, Canada; 3 Division of Neurology, Department of Medicine, University of Toronto, Toronto, Ontario, Canada; Hirosaki University Graduate School of Medicine, JAPAN

## Abstract

**Objective:**

*In vivo* Corneal Confocal Microscopy (IVCCM) is a validated, non-invasive test for diabetic sensorimotor polyneuropathy (DSP) detection, but its utility is limited by the image analysis time and expertise required. We aimed to determine the inter- and intra-observer reproducibility of a novel automated analysis program compared to manual analysis.

**Methods:**

In a cross-sectional diagnostic study, 20 non-diabetes controls (mean age 41.4±17.3y, HbA1c 5.5±0.4%) and 26 participants with type 1 diabetes (42.8±16.9y, 8.0±1.9%) underwent two separate IVCCM examinations by one observer and a third by an independent observer. Along with nerve density and branch density, corneal nerve fibre length (CNFL) was obtained by manual analysis (CNFL_MANUAL_), a protocol in which images were manually selected for automated analysis (CNFL_SEMI-AUTOMATED_), and one in which selection and analysis were performed electronically (CNFL_FULLY-AUTOMATED_). Reproducibility of each protocol was determined using intraclass correlation coefficients (ICC) and, as a secondary objective, the method of Bland and Altman was used to explore agreement between protocols.

**Results:**

Mean CNFL_Manual_ was 16.7±4.0, 13.9±4.2 mm/mm^2^ for non-diabetes controls and diabetes participants, while CNFL_Semi-Automated_ was 10.2±3.3, 8.6±3.0 mm/mm^2^ and CNFL_Fully-Automated_ was 12.5±2.8, 10.9 ± 2.9 mm/mm^2^. Inter-observer ICC and 95% confidence intervals (95%CI) were 0.73(0.56, 0.84), 0.75(0.59, 0.85), and 0.78(0.63, 0.87), respectively (p = NS for all comparisons). Intra-observer ICC and 95%CI were 0.72(0.55, 0.83), 0.74(0.57, 0.85), and 0.84(0.73, 0.91), respectively (p<0.05 for CNFL_Fully-Automated_ compared to others). The other IVCCM parameters had substantially lower ICC compared to those for CNFL. CNFL_Semi-Automated_ and CNFL_Fully-Automated_ underestimated CNFL_Manual_ by mean and 95%CI of 35.1(-4.5, 67.5)% and 21.0(-21.6, 46.1)%, respectively.

**Conclusions:**

Despite an apparent measurement (underestimation) bias in comparison to the manual strategy of image analysis, fully-automated analysis preserves CNFL reproducibility. Future work must determine the diagnostic thresholds specific to the fully-automated measure of CNFL.

## Introduction

Diabetic sensory polyneuropathy (DSP) is characterized by progressive, symmetrical and length-dependent loss of function to peripheral nerves resulting at least partially from chronic hyperglycemia. [1] DSP is estimated to be present in 50% of patients with type 1 diabetes (T1DM), and the presence of DSP exaggerates the risk of ulceration, infection and amputation. [2] Currently there is no effective disease-modifying treatment for DSP and intervention is aimed primarily at controlling the intensity and duration of hyperglycemic exposure. However, the selection of patients at the highest clinical need for intensified glycemic intervention could be informed by objective evidence for the presence of early neuropathy. [2]

Accurate diagnosis of DSP requires identifying abnormal electrodiagnostic data in combination with neuropathic signs and symptoms. [3, 4] This definition correlates well with large-fibre dysfunction and is thus effective at detecting the later stages of neuropathy. Identification of the earliest process of peripheral nerve injury requires the existence of a reliable and valid test for detecting damage to small, thinly- and un-myelinated nerve fibres as damage to these fibres precedes large fibre impairment. [5–7] The gold-standard for measuring small fibre neuropathy is the morphological assessment of intra-epidermal nerve fibres through skin biopsies, but its invasive nature makes it less practical for routine screening purposes. [8] To fill this gap, *in vivo* corneal confocal microscopy (IVCCM) has emerged as a tool for detecting early morphological alterations in the small nerve fibres, sampled non-invasively by imaging the nerve fibre plexus contained in the transparent cornea of the eye. These fibres arise from the ophthalmic division of the trigeminal nerve. Their morphological features correlate well with those in the intra-epithelial layer of skin, and cross-sectional and longitudinal studies of their diagnostic performance for DSP have for the most part been validated. [9–20] However, utility is substantially limited by the time and expertise required for image analysis.

To address this need, investigators from the University of Manchester have developed a tool capable of automated analysis of individual images, which holds the promise of eliminating the need for trained image analyst personnel and reducing the time that it takes to analyse a single image from 10–20 minutes to several seconds. [21] Ultimately, knowledge translation of IVCCM into clinical practice requires assurance of its reliability in the context of the complex methodologically-sound studies designed to appropriately evaluate reproducibility.

By way of an existing image repository from a previous detailed reproducibility study, in which each participant was repeatedly examined three times in a single day, we aimed to systematically determine the inter- and intra-observer reproducibility of a semi- and fully-automated image analysis protocol compared to that of the existing manual analysis reference standard. As a secondary objective, we aimed to explore correlation and agreement between protocols. [22]

## Methods

### Study Participants

Twenty-six type 1 diabetes participants and twenty non-diabetes controls, previously examined in a study of reproducibility between 2008 and 2010, were randomly selected from The Toronto Neuropathy Cohort study. Details of the nested study and the cohort study have previously been described. [22] In brief, participants with type 1 diabetes and non-diabetes controls were recruited from Endocrinology and Neurology clinics at the University Health Network, Toronto, Ontario, Canada. Controls were ascertained from spouses, family and friends of these participants. Inclusion required being 18 years of age or greater, and the absence of non-diabetes neuropathy. The study protocol was approved by the University Health Network research ethics board (approval number 08-0717-A), all participants provided written informed consent and the study was conducted in accordance with the Declaration of Helsinki.

### Clinical and Electrophysiological Variables

All participants underwent clinical evaluation, electrophysiological testing, and laboratory testing on the same day. As per research guidelines, DSP case definition required presence of at least one electrophysiological parameter abnormality in each of the peroneal and sural nerves, and the presence of one neuropathic sign or symptom. [3, 22–24] Electrophysiological parameters included amplitude potential and conduction velocity of the peroneal and sural nerve as well as the f-wave latency for the peroneal nerve. Age- and height-adjusted criteria for sural and peroneal parameters were applied and each parameter was scored as normal or abnormal based on laboratory reference values. [25] Research staff conducting the electrophysiological and clinical exams were blinded to the participants’ IVCCM results. All testing procedures were done in accordance with the standards of the American Association for Neuromuscular and Electrodiagnostic Medicine as well as the Canadian Society of Clinical Neurophysiology as previously described for this cohort. [26]

### In Vivo Corneal Confocal Microscopy Procedure

We performed bilateral examinations of the nerve plexus adjacent to the corneal Bowman’s layer using the Rostock cornea module of the Heidelberg Tomograph III using the 300μm field of view lens (Heidelberg Engineering, Smithfield, RI, USA) according to previously-published methods. [22, 23, 27] In brief, the technician administered a topical anaesthetic to the participant’s eye to temporarily suppress the corneal blink reflex. Then, a gel was used to form a bridge between the cornea and the sterile cap of the microscope lens. A 670 nm red wavelength diode laser illuminated the ocular structures and aided the technician in targeting the cornea’s apex. The volume scan mode was used to automatically capture 40 0.3 by 0.3 mm images that spanned a total depth of 50 μm at 1.3 μm increments. This was done twice per eye and then repeated on the contralateral eye. One examination yielded 80 images per eye (160 images in total), and each participant underwent three examinations, two by a single examiner and a third by an independent examiner. All examinations were done within a three hour period of each other. This allowed for systematic evaluation of both inter- and intra-observer reproducibility. Three distinct protocols for quantifying IVCCM parameters were then applied: manual image analysis, semi-automated image analysis and fully-automated image analysis.

#### Manual Image Analysis

A research technician visually examined the 80 images obtained per eye and selected the image which had the highest density of nerve fibres and was of high technical quality with respect to focus and contrast. This was repeated for the participant’s other eye such that a total of two images were selected per participant. The selected images were then analysed using CCMetrics, developed by the University of Manchester group. This involved manually tracing and placing cursor marks over the fibres and branches with a graphic pen tablet which allowed for quantification of corneal nerve fibre length (CNFL, measured in units of mm/mm^2^), corneal nerve fibre density (CNFD, measured in units of fibres/mm^2^) and corneal nerve branch density (CNBD, measured in units of branches/mm^2^). [28] Nerve branches included all branches from main nerve fibres, and any subsequent branches attached to those. The CNFL, CNFD and CNBD obtained from both eyes were averaged to determine the participant’s overall corneal nerve parameters. We note that the results of a reproducibility analysis using this manual protocol have been previously published, and we present them as a means of comparison with the new methods described below.

#### Semi-Automated Image Analysis

In the semi-automated analysis protocol the two images per participant were selected in the same way as they were selected in the manual image analysis protocol. Parameter quantification was accomplished using ACCMetrics, an automated software for IVCCM image analysis also developed the University of Manchester group. [21, 29] ACCMetrics detects low contrast nerve fibres amongst image noise. The software took approximately 15 seconds to analyse one image and unlike CCMetrics, required no preliminary, manual image tracing. ACCMetrics was used to analyse the two images chosen per participant, and the overall CNFL, CNFD and CNBD were obtained by averaging the results of these two images.

#### Fully-Automated Image Analysis

A research technician visually examined the two sets of 40 images obtained per eye, selecting one set for analysis. Within the selected set, the images (between 5 and 15) that included the nerve plexus layer were selected by the technician and all were analysed by ACCMetrics. The image with the highest CNFL value was chosen per eye and the results were averaged to obtain the participant’s overall CNFL, CNFD and CNBD.

### Statistical Methods

Statistical analysis was performed using SAS 9.2 (SAS Institute, Cary, NC). Baseline characteristics of the non-diabetes controls, participants with type 1 diabetes without DSP, and participants with type 1 diabetes with DSP were compared using the χ^2^-test or Fisher's exact test (for smoking prevalence), and one way ANOVA (for continuous variables). Inter- and intra-observer reproducibility of each IVCCM parameter was assessed using the intraclass correlation coefficients ICC(2,1), as per the notation Shrout and Fleiss. [30] As described in our previous work, [22] the ICC(2,1) was used since it produces less biased and more conservative estimates of reproducibility and reliability compared to the other classes described by Shrout and Fleiss. This method also has high generalizability as it assumes that all participants are rated by the same raters from a random subset of the population of all possible raters. [22] ICC >0.80 were considered very good, 0.61–0.80 good, 0.41–0.60 moderate, 0.21–0.40 fair, and ICC <0.21 poor. [31] ICC were compared using their 95% confidence intervals (CI). [32] We performed two stratified analyses to explore the reproducibility within subgroups of the cohort: The first was an evaluation of ICC within the subgroup with DSP, and the second was an evaluation of the subgroup with CNFL values below 12.3mm/mm^2^, a threshold that represents the 2.5th percentile of the distribution observed in a previous study of non-diabetes controls using the manual analysis protocol. [33] This threshold approximates the lower extreme of the normal CNFL distribution values reported in a second, independent publication. [34] As a secondary objective, correlation and agreement between the protocols were assessed using Pearson correlation coefficients and the method of Bland & Altman, respectively. [35] Both absolute and percentage differences were used in the calculation of these metrics, and the 95%CI of the differences were reported. An alpha level of 0.05 was used for all comparisons.

## Results

Clinical characteristics of the 46 study participants categorized as non-diabetes controls (n = 20), diabetes without DSP (n = 13), and diabetes with DSP (n = 13) are shown in Table 1. Broad variability of clinical factors associated with risk of DSP was observed between groups. These included age (p<0.001), diabetes duration (p<0.001) and HbA1c (p<0.001), all of which were highest in the group with both diabetes and DSP. Furthermore, broad variability was also observed in objective measures of DSP severity, such as sural nerve amplitude, which ranged from 17.3±7.7 μv in the non-diabetes controls to 2.3±1.8 μv in the group with both diabetes and DSP (p<0.001), showing significant nerve function impairment in the diabetes with DSP group. Collectively, these findings indicate a broad spectrum of DSP severity across the study group. Similarly, broad variability was also observed in the IVCCM parameters. CNFL_manual_ was 16.2±3.96 mm/mm^2^ in non-diabetes controls, and 16.9±3.79 mm/mm^2^ in participants with type 1 diabetes without DSP, but substantially lower at 12.0±4.23 mm/mm^2^ for diabetes particpants with DSP. Similar patterns were observed for CNFD_Manual_ and CNBD_Manual_, though differences between groups did not reach statistical significance for the CNBD_Manual_ parameter. However, we noted differences in the mean values between the manual, semi-automated and fully-automated values. Specifically, for all variables, the mean values of CNFL, CNFD and CNBD determined by the semi-automated and fully-automated protocols appeared to be substantially lower than the manual measurements.

**Table 1:** Baseline clinical and biochemical characteristics of the 46 participants according to diabetes and DSP status.

	Non-diabetes controls (n = 20)††	Participants with type 1 Diabetes without DSP (n = 13)	Participants with type 1 Diabetes with DSP (n = 13)	P value
*Clinical Characteristics*				
Male sex, n (%)*	5 (25%)	11 (85%)	8 (61%)	0.009
Age (years)	41.3±17.3	30.3±13.7	56.2±8.7	<0.001
Duration of Type 1 diabetes (years)	-	10.7±6.2	34.8±13.0	<0.001
Current/past smoker†	6(30%)	0(0%)	2(15%)	0.070
Systolic Blood Pressure (mmHg)	127±14	122±15	140±20	0.021
Diastolic Blood Pressure (mmHg)	79±10	70±10	73±6	0.025
HbA1c (%)	5.5±0.4	7.5±1.3	8.5±2.2	<0.001
Total Cholesterol (mmol/L)	5.5±0.9	4.7±0.8	3.9±0.8	<0.001
Nerve Conduction Data				
Sural Nerve Amplitude Potential (μV)	17.3±7.7	14.3±5.1	2.3±1.8	<0.001
Sural Nerve Conduction Velocity (m/s)	52.7±4.3	47.9±2.8	40.0±4.9	<0.001
Peroneal Nerve Amplitude Potential (mV)	7.1±1.9	7.4±3.3	1.6±1.7	<0.001
Peroneal Nerve Conduction Velocity (m/s)	48.4±2.7	44.9±2.3	33.4±6.8	<0.001
Peroneal Nerve F-Wave (ms)	46.4±3.0	50.7±4.6	68.6±10.1	<0.001
IVCCM Parameters				
CNFL_Manual_ (mm/mm^2^)	16.2±3.96	16.9±3.79	12.0±4.23	0.006
CNFL_Semi-Automated_ (mm/mm^2^)	10.2±3.38	9.74±3.56	7.48±2.15	0.053
CNFL_Fully-Automated_ (mm/mm^2^)	12.53±2.87	12.2±3.19	9.58±2.19	0.013
CNFD_Manual_ (fibres/mm^2^)	40.3±9.34	40.6±9.46	30.3±10.54	0.011
CNFD_Semi-Automated_ (fibres/mm^2^)	22.77±12.47	20.94±11.37	11.54±6.60	0.017
CNFD_Fully-Automated_ (fibres/mm^2^)	28.06±8.55	27.77±8.49	17.52±8.13	0.002
CNBD_Manual_ (branches/mm^2^)	31.39±10.24	28.21±11.67	22.22±12.21	0.083
CNBD_Semi-Automated_ (branches/mm^2^)	17.22±16.01	8.97±9.22	7.69±9.50	0.073
CNBD_Fully-Automated_ (branches/mm^2^)	24.99±12.16	19.23±16.76	13.25±13.13	0.068

One way ANOVA unless otherwise specified

*Chi-square test

† Fisher’s exact test

†† Values presented in column 1 (other than for Semi-Automated and Fully-Automated CCM parameters) have been presented in a previous publication and are shown here for comparison with the diabetes subgroups.

IVCCM, in-vivo corneal confocal microscopy; CNFL, corneal nerve fibre length; CNFD, corneal nerve fibre density; CNBD, corneal nerve branch density.

For the primary objective of determining reproducibility in all participants, we present the results of the inter- and intra-observer reliability analysis in Table 2. The first row of data in this table shows the inter-observer ICC for CNFL_Manual_, CNFL_Semi-Automated_ and CNFL_Fully-Automated_ and the p-values for their comparison. Specifically, the respective values were 0.73, 0.75 and 0.78, and none of the three-way comparisons between these ICC differed significantly. The second row of data in this table reports the intra-observer ICC for CNFL_Manual_, CNFL_Semi-Automated_ and CNFL_Fully-Automated_, which were 0.72, 0.73 and 0.84 respectively. The intra-observer reproducibility of CNFL_Fully-Automated_ was larger than that of CNFL_Manual_ (p = 0.021) and CNFL_Semi-Automated_ (p = 0.039).

**Table 2:** Intra Class Correlation coefficients for all participants (n = 46).

	Observer reliability measure	The manual protocol ICC and 95%CI*	The semi-automated protocol ICC and 95%CI	Comparison of semi-auto to manual P-Value	The fully automated protocol ICC and 95%CI	Comparison of fully auto to manual P-Value	Comparison of fully auto to semi-auto P-Value
CNFL†	Inter	0.73(0.55,0.84)	0.75(0.58,0.86)	0.39	0.78(0.34,0.91)	0.40	0.46
	Intra	0.72(0.50,0.85)	0.73(0.56,0.84)	0.43	0.84(0.73,0.91)	0.021	0.039
CNFD	Inter	0.61(0.39,0.76)	0.68(0.48,0.81)	0.68	0.50(0.17,0.71)	0.83	0.95
	Intra	0.57(0.33,0.73)	0.60(0.39,0.76)	0.37	0.63(0.41,0.78)	0.27	0.38
CNBD	Inter	0.32(-0.07,0.61)	0.40(0.13,0.61)	0.28	0.50(0.23,0.69)	0.093	0.23
	Intra	0.61(0.40,0.77)	0.31(0.02,0.55)	0.99	0.43(0.16,0.64)	0.95	0.18

ICC: Intra-class correlation coefficient (2, 1). Inter refers to inter-observer reproducibility, intra to intra-observer reproducibility. The manual protocol included manual image selection and manual image analysis. The semi-automated protocol included manual image selection and automated analysis. The fully-automated protocol included automated image selection and automated analysis. CNFL, corneal nerve fibre length. CNFD, corneal nerve fibre density. CNBD, corneal nerve branch density.

* Manual protocol ICC have been presented in a previous publication and are shown here for comparison.

† For the 11-member sub-population with CNFL below 12.3mm/mm^2^, the inter-observer ICC for CNFL_Manual_, CNFL_Semi-Automated_, and CNFL_Fully-Automated_ were 0.54(-0.09,0.85), 0.51(-0.04,0.83), and 0.73(0.25,0.92), respectively. P-values for comparison of the ICC of CNFL_Semi-Automated_ to CNFL_Manual_, CNFL_Fully-Automated_ to CNFL_Manual_, and CNFL_Fully-Automated_ to CNFL_Semi-Automated_ were 0.56, 0.17, and 0.15, respectively. The corresponding intra-observer ICC for CNFL_Manual_, CNFL_Semi-Automated_, and CNFL_Fully-Automated_ were 0.74(0.26,0.92), 0.68(0.20,0.90), and 0.86(0.55,0.96), respectively. The p-values for the corresponding comparisons were 0.64, 0.16, and 0.19.

The third and fourth rows of the table display the inter- and intra-observer ICC of CNFD_Manual_ CNFD_Semi-Automated_ and CNFD_Fully-Automated_. All CNFD ICC fell in the ranges of good or moderate with no significant differences between protocols. The fifth and sixth rows display the inter- and intra-observer ICC of CNBD_Manual_, CNBD_Semi-Automated_ and CNBD_Fully-Automated_. These ranged between good and fair and again no significant differences between protocols were observed.

Stratified analyses by diabetes-status are presented in Tables 3 and 4. These revealed that the ICC measured were similar between the non-diabetes controls and the participants with T1DM. When we sub-divided the type 1 diabetes participants into those with and without DSP, similar ICC for CNFL were observed (described in Table 4 Legend). Additionally, reproducibility was studied in the sub-population with CNFL below 12.3mm/mm^2^; 11(24%) of the entire study population had CNFL in this range. For this subgroup, the inter-observer ICC for CNFL_Manual_, CNFL_Semi-Automated_, and CNFL_Fully-Automated_ was 0.54(-0.09,0.85), 0.51(-0.04,0.83), and 0.73(0.25,0.92), respectively, while the intra-observer ICC was 0.74(0.26,0.92), 0.68(0.20,0.90), and 0.86(0.55,0.96) (also summarized in Table 2, Legend). In this subgroup of individuals with the lowest CNFL values, inter- and intra-observer ICC were moderate and good, respectively, for both CNFL_Manual_ and CNFL_Semi-Automated_, and all lower than that observed for the ICC in the entire study population. However, ICC for CNFL_Fully-Automated_ were in keeping with the levels observed in the entire study population. None of the ICC values for each of CNFL_Manual_, CNFL_Semi-Automated_, and CNFL_Fully-Automated_ differed significantly (p values shown in Table 2, Legend).

**Table 3:** Intra Class Correlation coefficients for all non-diabetes controls (n = 20).

	Observer reliability measure	The manual protocol ICC and 95%CI*	The semi-automated protocol ICC and 95%CI	Comparison of semi-auto to manual P-Value	The fully automated protocol ICC and 95%CI	Comparison of fully auto to manual P-Value	Comparison of fully auto to semi-auto P-Value
CNFL	Inter	0.68(0.35,0.86)	0.73(0.43,0.88)	0.34	0.75(0.25,0.91)	0.38	0.50
	Intra	0.72(0.39,0.88)	0.77 (0.50,0.90)	0.32	0.81(0.59,0.92)	0.16	0.31
CNFD	Inter	0.39(-0.04,0.70)	0.72(0.42,0.88)	0.018	0.52(0.09,0.78)	0.27	0.92
	Intra	0.51(0.10,0.78)	0.69(0.36,0.87)	0.11	0.72(0.39,0.88)	0.084	0.41
CNBD	Inter	0.35(-0.11,0.71)	0.44(0.04,0.73)	0.31	0.50(0.08,0.77)	0.23	0.39
	Intra	0.58(0.21,0.81)	0.58(0.08,0.75)	0.74	0.49(0.09,0.76)	0.70	0.47

ICC: Intra-class correlation coefficient (2, 1). Inter refers to inter-observer reproducibility, intra to intra-observer reproducibility. The manual protocol included manual image selection and manual image analysis. The semi-automated protocol included manual image selection and automated analysis. The fully-automated protocol included automated image selection and automated analysis. CNFL, corneal nerve fibre length. CNFD, corneal nerve fibre density. CNBD, corneal nerve branch density.

* Manual protocol ICC have been presented in a previous publication and are shown here for comparison.

**Table 4:** Intra Class Correlation coefficients for all T1DM participants (n = 26).

	Observer reliability measure	The manual protocol ICC and 95%CI*	The semi-automated protocol ICC and 95%CI	Comparison of semi-auto to manual P-Value	The fully automated protocol ICC and 95%CI	Comparison of fully auto to manual P-Value	Comparison of fully auto to semi-auto P-Value
CNFL†	Inter	0.75(0.51,0.88)	0.75(0.53,0.88)	0.52	0.79(0.32,0.92)	0.44	0.44
	Intra	0.72(0.44,0.87)	0.68(0.40,0.84)	0.66	0.84(0.68,0.92)	0.054	0.023
CNFD	Inter	0.72(0.46,0.86)	0.58(0.27,0.79)	0.88	0.42(0.05,0.69)	0.99	0.42
	Intra	0.59(0.27,0.79)	0.41(0.05,0.68)	0.89	0.53(0.21,0.76)	0.53	0.21
CNBD	Inter	0.22(-0.09,0.52)	0.19(-0.22,0.54)	0.56	0.45(0.10,0.70)	0.091	0.068
	Intra	0.61(0.29,0.80)	0.16(-0.21,0.51)	>0.99	0.34(-0.06,0.64)	0.34	0.18

ICC: Intra-class correlation coefficient (2, 1). Inter refers to inter-observer reproducibility, intra to intra-observer reproducibility. The manual protocol included manual image selection and manual image analysis. The semi-automated protocol included manual image selection and automated analysis. The fully-automated protocol included automated image selection and automated analysis. CNFL, corneal nerve fibre length. CNFD, corneal nerve fibre density. CNBD, corneal nerve branch density.

* Manual protocol ICC have been presented in a previous publication and are shown here for comparison.

† For the 13-member sub-population without DSP, inter-observer ICC for CNFL_Fully-Automated_ was 0.78(0.30,0.93) and intra-observer ICC for CNFL_Fully-Automated_ was 0.84(0.55,0.95). For the 13-member sub-population with DSP, the inter-observer ICC for CNFL_Fully-Automated_ was 0.65(0.05,0.89) and intra-observer ICC for CNFL_Fully-Automated_ was 0.72(0.31,0.90). Comparisons of these ICC were not statistically significant.

**Fig 1:**
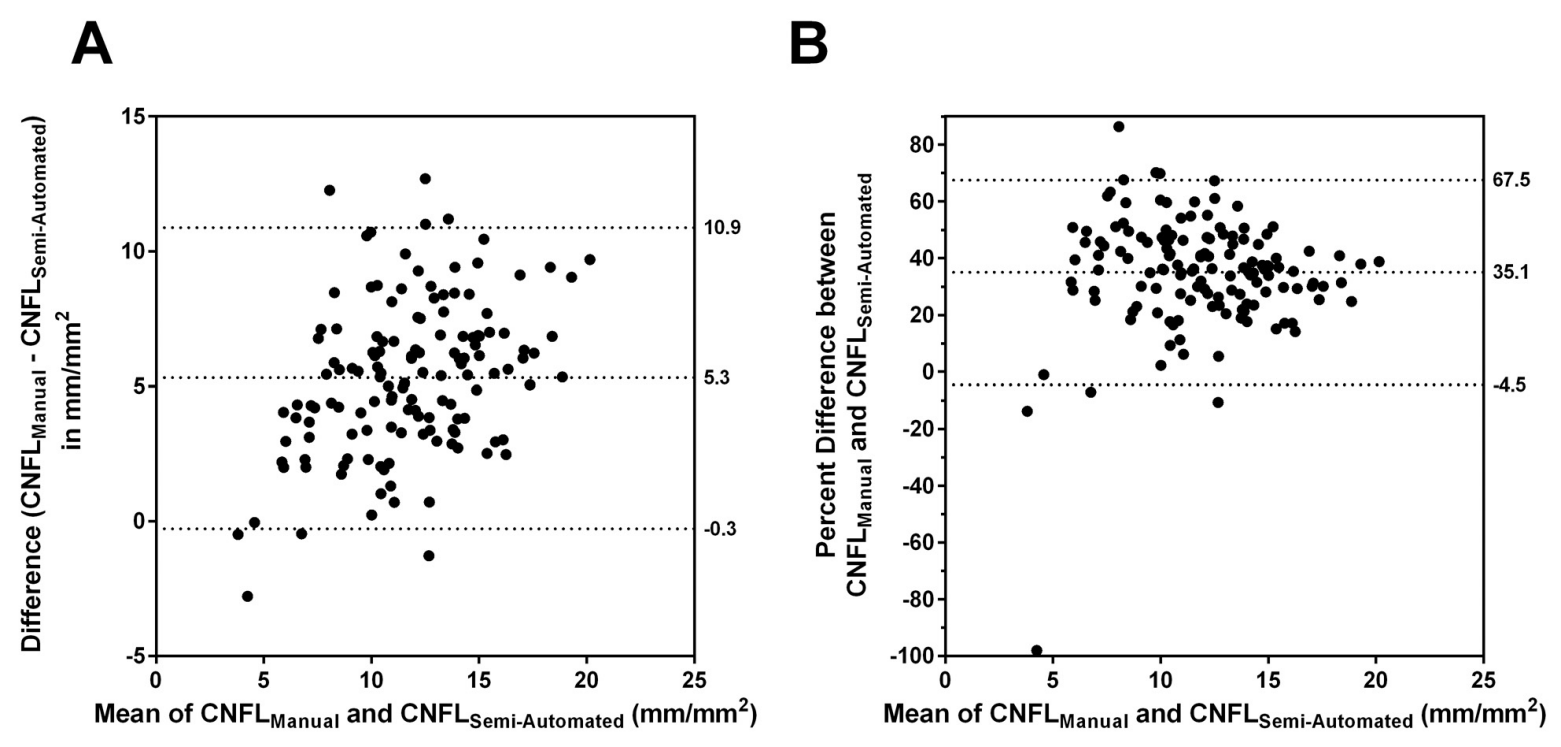
Agreement between CNFL_Manual_ and CNFL_Semi-Automated_. In Panels A and B, the reference lines, from top to bottom, denote the 97.5th percentile, the mean, and the 2.5th percentile for the indicated differences.

To explore the underestimation of the semi-automated and fully-automated IVCCM measures observed in the final section of Table 1, we determined the correlation and agreement between these protocols as part of a secondary objective. Fig 1 presents the Bland-Altman plots of agreement between CNFL_Manual_ and CNFL_Semi-Automated_. While the two protocols correlated well (Pearson r = 0.72), there was a marked lack of agreement. Fig 1 Panel A plots the difference between CNFL_Manual_ and CNFL_Semi-Automated_ against the average of these variables, with reference lines, from top to bottom, denoting the 97.5^th^ percentile, the mean, and the 2.5^th^ percentile of the differences. On average, the semi-automated protocol underestimated the manual protocol by 5.3 [95% CI (-0.3, 10.9)] mm/mm^2^. Fig 1 Panel A also shows that the magnitude of underestimation increased as the mean of CNFL_Manual_ and CNFL_Semi-Automated_ increased. Fig 1 Panel B shows the same Bland-Altman plot but with the difference between CNFL_Manul_ and CNFL_Semi-Automated_ expressed as a percentage of CNFL_Manual_. This shows that on average, CNFL_Semi-Automated_ underestimated CNFL_Manual_ by 35.1 [95% CI (-4.5, 67.5)] %.

**Fig 2:**
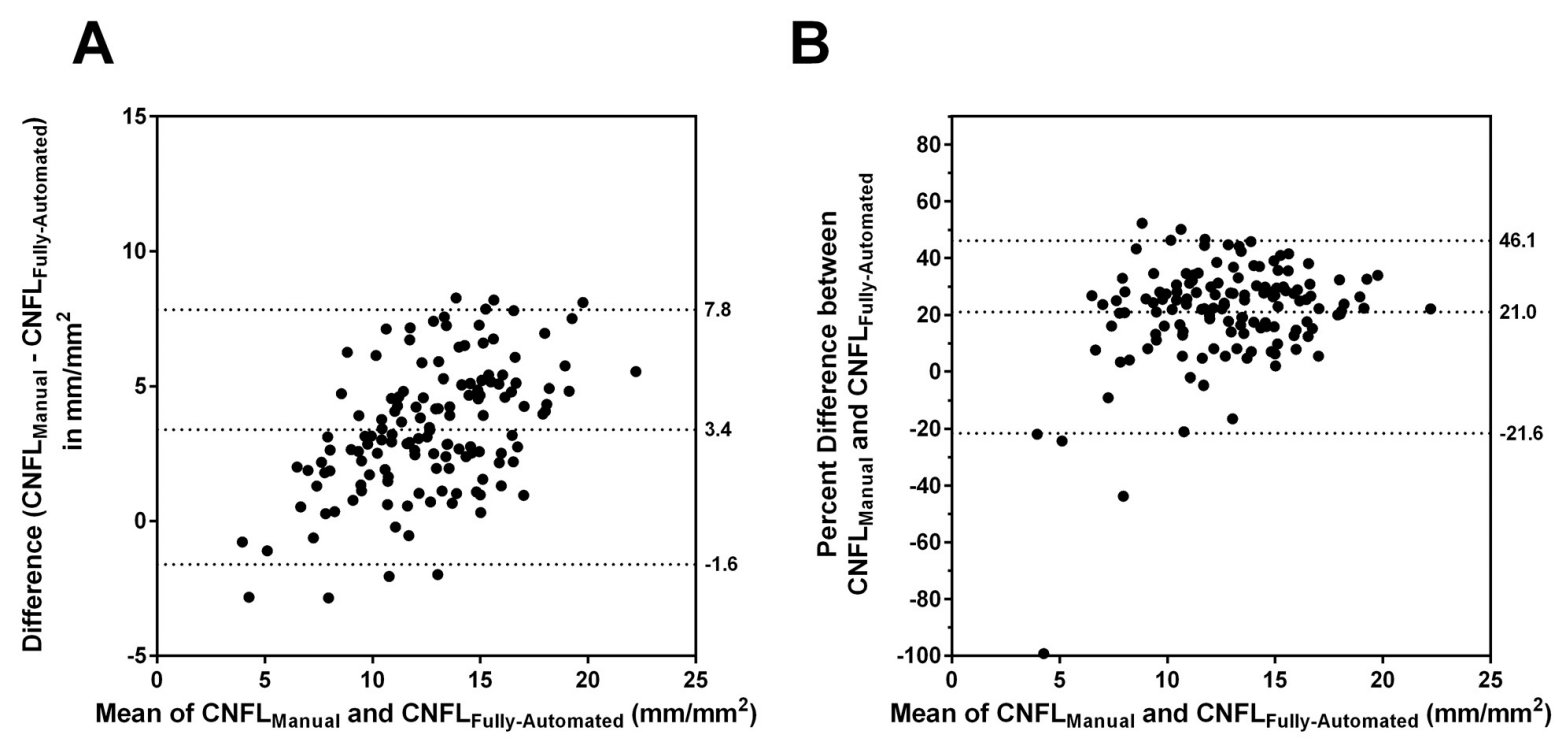
Agreement between CNFL_Manual_ and CNFL_Fully-Automated_. In Panels A and B, the reference lines, from top to bottom, denote the 97.5th percentile, the mean, and the 2.5th percentile for the indicated differences.

Bland-Altman plots of agreement between CNFL_Manual_ and CNFL_Fully-Automated_ are shown in Fig 2. There was excellent correlation between the protocols (Pearson r = 0.82), but Fig 2 Panel A shows that on average, CNFL_Fully-Automated_ underestimated CNFL_Manual_ by 3.4 [95% CI (-1.6, 7.8)] mm/mm^2^. Fig 2 Panel B shows that this was equivalent to a 21 [95% CI (-21.6, 46.1)] % underestimation.

## Discussion

We studied, on three independent examinations, 46 participants representing a broad spectrum of nerve injury to determine the inter- and intra- observer reproducibility of a fully- and semi-automated protocol of IVCCM image analysis and compared them to the reference manual protocol. Though our main objective of this analysis was to show that the automated approach yields a level of reproducibility no worse than the previously studied manual approach, we did observe three key findings that add to the understanding of IVCCM for diabetic neuropathy. First, we report that the levels of inter- and intra- observer reproducibility were generally similar and good (ICC in the range of 0.6 to 0.8) between manual and automated image selection protocols, noting a superior reproducibility of the CNFL_Fully-Automated_ approach that was limited to intra-observer reproducibility. Second, we confirmed that the poorer reproducibility of the other IVCCM parameters–CNFD and CNBD–are not overcome by automated analysis and remain insufficient in comparison to CNFL. Finally, in our secondary analysis of agreement, we observed systematic underestimation of IVCCM parameters as measured by the fully- and semi-automated protocols compared to the reference manual protocol.

The finding of similar reproducibility of IVCCM parameters between automated and manual protocols is of fundamental importance for the translation of this technology as a measure of neuropathy to clinical trials and clinical practice. Specifically, the feasibility of the manual approach is limited by major demands on technician time for image analysis, and this limitation is overcome by the automated protocols. Though several studies have demonstrated that the reproducibility of performing repeat manual analysis of the same IVCCM image, described as “image-level reproducibility”, is very good in that ICC generally exceed 0.8, [22, 36, 37] such investigative test methodology does not sufficiently represent the true clinical reliability of the procedure. The more sound “study-level reproducibility” involves the analysis of images obtained from repeated patient IVCCM examinations and is a more clinically relevant measure of reproducibility as it accounts for the variation inherent in conducting multiple examinations. Such study-level reproducibility was later reported for the manual analysis technique, which revealed ICC levels in the good to very good range (exceeding levels of 0.7) [22, 37, 38]; quantification of nerves in the inferior whorl region of the cornea has also shown good study-level reproducibility. [39] However, to date, only the image-level reproducibility for automated image analysis has been reported (ICC level 1.0 indicating perfect reproducibility). [29] The current analysis represents the first confirmation of acceptable study-level reproducibility obtained from automated image analysis protocols using an image repository created through meticulous re-examination of participants by blinded examiners. The ICC that we report represent an unbiased estimate of the clinical reproducibility of automated IVCCM procedures. We thus confirm that the clinically-relevant inter- and intra-observer reliability of CNFL is preserved using automated approaches compared to the reference standard manual examination technique.

In contrast to these very good levels of reproducibility for CNFL, we were able to confirm that automated image analysis did not overcome the previously-described limitations in the inter- and intra-observer reproducibility of CNFD and CNBD. [22, 36, 37] We had previously hypothesized that the distinction between nerve fibres and nerve branches is not consistently clear in the examination of separate images—specifically, two crossing fibres might be interpreted as a single branching fibre but subsequently interpreted as two fibres without branches. For this reason, we had concluded that the reproducibility of these measures was inherently impaired for manual analysis protocols. [22] Though the automated image analysis protocols may remove image-level decision bias, the current results demonstrate that such bias is not reconciled by automation when applied in the context of the more clinically-relevant study-level analysis of independent clinical examinations. Inherently, CNFL is less susceptible to this measurement bias as it represents an integrated measure of the length of all nerve fibres and branches in the microscope field. As reproducibility is a key characteristic in the hierarchical model for studying diagnostic performance, [40] the current analysis confirmed the inherent advantage of CNFL over the other parameters and supports it as the preferred candidate IVCCM parameter.

**Fig 3:**
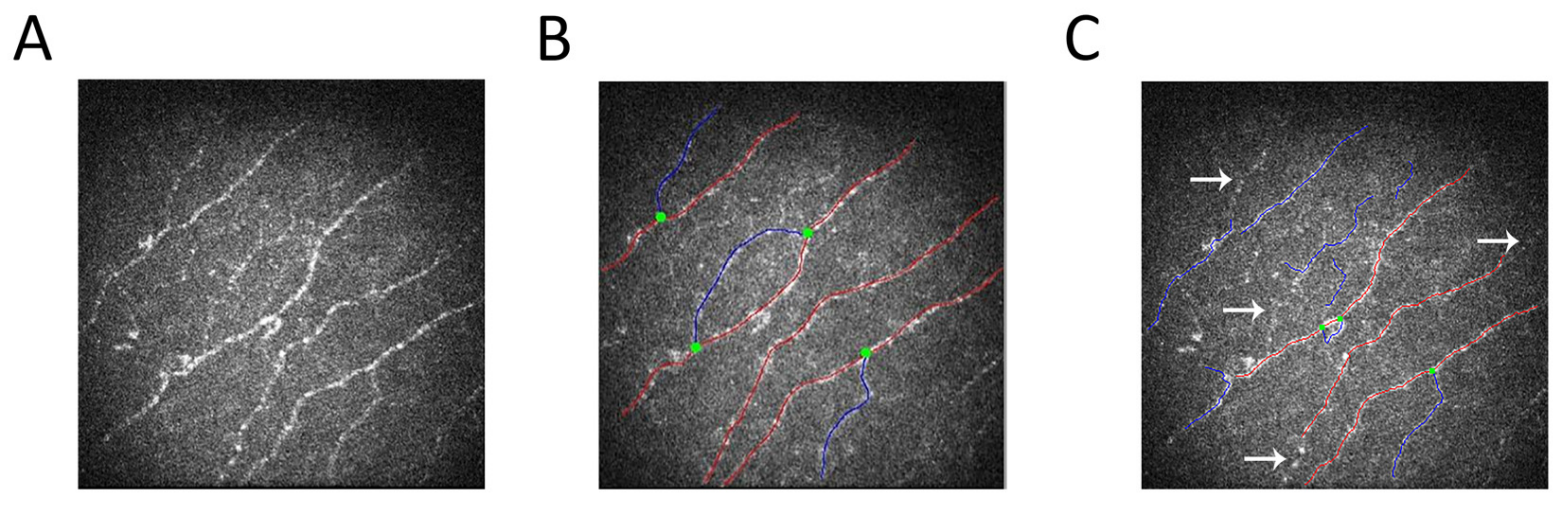
Illustration of Underestimation Measurement Bias by the Automated Image Analysis Protocol. A) IVCCM image obtained using the Rostock corneal module of the Heidelberg Tomograph III using the 300 μm field of view lens B) Image from panel A traced for parameter quantification using CCMetrics, the software used in the manual protocol of IVCCM image analysis. Red lines indicate fibres, blue lines indicate branches and green dots indicate branching points. C) Image from Panel A post-analysis by ACCMetrics, the software used in automated IVCCM image analysis, showing which nerves were captured by the automated quantification process. The arrows were included to indicate nerves that were not quantified by ACCMetrics despite being traced for quantification using the manual protocol. This inability of ACCMetrics to detect fainter nerve fibres is representative of the entire dataset and explains the measurement (underestimation) bias inherent in using automated IVCCM image analysis protocols.

Though not the primary objective of this analysis, we explored correlation and agreement for CNFL between the three image analysis protocols. Although the correlation between the manual and fully-automated protocols were excellent (Pearson correlation coefficient 0.82), we report a substantial measurement bias. Specifically, the fully-automated approach was associated with average underestimation of 3.4 mm/mm^2^, which corresponded to 21% underestimation. An even greater degree of measurement bias has previously been reported with an earlier version of the automated software. [28] We observed that the underestimation of CNFL by both automated protocols arose as a result of an inability of the automated software to detect the fainter, lower contrast nerve fibres against background structures (see Fig 3 as a representative example). As the automated protocols offered overwhelming practical advantages over manual techniques in terms of time and resources required for image analysis, focused research is urgently required to determine the diagnostic thresholds for detecting DSP that are unique to the efficient automated protocols.

Although this study of clinically-relevant reproducibility had major methodological advantages over previous investigative test research for IVCCM, we acknowledge potential limitations. First, though we previously found excellent agreement between lens type, [41] we used a 300 μm lens that produced 0.3 x 0.3 mm^2^ field of view images, while other investigators have more commonly used a 400 μm lens that produces a 0.4 x 0.4 mm^2^ field of view. Second, there are minor variations in the protocol for image acquisition as compared to other study groups in that we used the “volume scan” mode [22, 23, 42] rather than the “section mode” [9, 29, 36, 38, 43, 44] for image acquisition and we implemented a protocol that used a single image per eye rather than multiple. [45] Third, we did not systematically determine analysis time in this study to further substantiate time- and resource-savings. Finally, our results may not be generalizable to patients with type 2 diabetes.

Automating the process of IVCCM image analysis has the potential to overcome an important barrier to the clinical implementation of this neuropathy biomarker–its resource and time consuming nature. Confirming the reproducibility of the semi- and fully-automated protocols was an important step towards verifying its appropriateness for clinical and investigative use. The measurement bias between the automated and the manual approaches requires verification in a larger cohort. Furthermore it is imperative to establish the diagnostic thresholds for the identification of diabetic neuropathy and its future risk that are specific to the automated protocols.

## Supporting Information

S1 File Relevant Data. This file includes all relevant data to support the results of the reproducibility analysis.
(XLSX)
